# Chlorosis

**Published:** 1901-05-25

**Authors:** 


### CHLOROSIS.

The point of view from which many of the phenomena of chlorosis are regarded has altered considerably during recent years, so that now again, as in that long ago when by giving the disorder such names as *febris amatoria*, *icterus amantium*, and so forth, physicians expressed their belief in its sexual origin, the tendency is to regard the disorder as associated in some way with the function of reproduction, perhaps, in fact, as but an exaggeration of certain changes which normally occur in woman preparatory to her great function of child-bearing. Time was when attention was principally fixed upon the changes in the blood in this disease. Poverty of blood was looked upon as the central fact to be regarded. Anæmia and debility being considered as almost of necessity coincident, and chlorosis being evidently a condition of anæmia, every effort was made to "build up" new blood.

Perhaps the microscope was responsible. At any rate, we can have no doubt that by depending too much upon the counting of blood corpuscles both in diagnosis and in estimating progress physicians have for many years past hovered on the verge of error.

Of late, however, with more accurate clinical methods at our disposal, doubt has been thrown upon much that was not so long ago considered certain. Speaking at a recent meeting of the British Balneological and Climatological Association Professor Clifford Allbutt pointed out that some of the tests on which we have relied can no longer be trusted. "For many years," he says, "we, or our clinical clerks for us, have been industriously engaged in making blood counts. Now of the value of blood counts in the more eccentric deviations of the blood from the standard of health I am at present not called upon to speak; we leave such perversions out of account. But even if we regard for the moment the red corpuscles only, as in chlorosis, for instance, the value of blood counts, if not depreciated beyond all usefulness, proves to be far less directly interpretive than we had supposed." He goes on to show that a drop of blood taken in the usual way is not by any means representative of the mass of the blood in the body, varying as it does according to the condition of the cutaneous circulation, according to the exercise taken or of mental work done, according to the time in relation to sleep, and in other ways.

Not only do we thus have it that the blood counts, on which so much has been made to hinge, are useless or misleading, but by new methods of research new factors are being introduced into the problem. More especially do we now have to consider the total quantity of blood present in the body. We are now told that the impoverished condition of the blood in chlorosis, which to some observers has appeared to be the main departure from health and sufficient to account for all the symptoms of the disease, is after all only apparent, that there are corpuscles enough and hæmoglobin enough, only that they are scattered through far too large a quantity of plasma—plasma which, useful as it may be for purposes of nutrition, is obviously of but small service in carrying oxygen to the tissues and in thus maintaining the functional activity of the body.

Taking this more recent view of chlorosis it would appear that the dyspnœa, the palpitation, and the not infrequent dilatation of the heart in chlorosis are to be explained, not by lack of blood, but by the fact that in this disease the bulk of the blood is increased without proportionate increase in the corpuscular element. Thus the therapeutical problem is not so much how to multiply the red corpuscles as how to diminish the plasma in which they are extended. "Pull the blood together and there will be corpuscles enough, the heart will not have to deliver excessive parcels of blood to the lungs and elsewhere, and its dilatation will recede in so far as it may have been due, not to malnutrition, but to need of greater temporary capacity." On this hypothesis many of the symptoms of chlorosis appear easy of explanation, and the somewhat mixed ideas which have long prevailed as to therapeutics seem to receive some degree of clarification. We cannot admit, however, that we are any nearer the prime cause and origin of the disease. Probably the old physicians were right, and certainly a considerable number of modern physicians are inclined to agree with them in thinking that sexuality has much to do with the matter, that this excessively rich blood plasma is in a sense a preparation for maternity, and that many of the symptoms which accompany the condition are but the organic expression of that deeply-rooted desire for maternity which, however little it may be shown, or even felt so far as the ordinary consciousness is concerned, is the motive power in the life of normal woman. This raises at once the ticklish question of marriage as a cure for chlorosis, about which we will only say that it is a wasteful thing to use a steam hammer to crack nuts. Still, the nuts do generally crack.

